# Functional network topology of the right insula affects emotion dysregulation in hyperactive-impulsive attention-deficit/hyperactivity disorder

**DOI:** 10.1038/s41598-021-94426-8

**Published:** 2021-07-22

**Authors:** Tammo Viering, Pieter J. Hoekstra, Alexandra Philipsen, Jilly Naaijen, Andrea Dietrich, Catharina A. Hartman, Jan K. Buitelaar, Andrea Hildebrandt, Carsten Gießing, Christiane M. Thiel

**Affiliations:** 1 Biological Psychology, Department of Psychology, School of Medicine and Health Sciences, Carl-von-Ossietzky Universität Oldenburg, Postfach 2503, 26111 Oldenburg, Germany; 2 Department of Child and Adolescent Psychiatry, University Medical Center Groningen, University of Groningen, Groningen, The Netherlands; 3 Department of Psychiatry and Psychotherapy, University of Bonn, Bonn, Germany; 4 Department of Cognitive Neuroscience, Donders Institute for Brain, Cognition and Behaviour, Radboud University Medical Center, Nijmegen, The Netherlands; 5 Centre for Cognitive Neuroimaging, Donders Institute for Brain, Cognition and Behaviour, Radboud University, Nijmegen, The Netherlands; 6 Department of Psychiatry, Interdisciplinary Center Psychopathology and Emotion Regulation, University Medical Center Groningen, University of Groningen, Groningen, The Netherlands; 7 Karakter Child and Adolescent Psychiatry University Centre, Nijmegen, The Netherlands; 8 Psychological Methods and Statistics, Department of Psychology, School of Medicine and Health Sciences, Carl-von-Ossietzky Universität Oldenburg, Oldenburg, Germany; 9 Research Center Neurosensory Science, Carl-von-Ossietzky Universität Oldenburg, Oldenburg, Germany; 10 Cluster of Excellence “Hearing4all”, Carl von Ossietzky Universität Oldenburg, Oldenburg, Germany

**Keywords:** Medical research, Emotion, Insula, Psychology, Developmental disorders

## Abstract

Emotion dysregulation is common in attention-deficit/hyperactivity disorder (ADHD). It is highly prevalent in young adult ADHD and related to reduced well-being and social impairments. Neuroimaging studies reported neural activity changes in ADHD in brain regions associated with emotion processing and regulation. It is however unknown whether deficits in emotion regulation relate to changes in functional brain network topology in these regions. We used a combination of graph analysis and structural equation modelling (SEM) to analyze resting-state functional connectivity in 147 well-characterized young adults with ADHD and age-matched healthy controls from the NeuroIMAGE database. Emotion dysregulation was gauged with four scales obtained from questionnaires and operationalized through a latent variable derived from SEM. Graph analysis was applied to resting-state data and network topology measures were entered into SEM models to identify brain regions whose local network integration and connectedness differed between subjects and was associated with emotion dysregulation. The latent variable of emotion dysregulation was characterized by scales gauging emotional distress, emotional symptoms, conduct symptoms, and emotional lability. In individuals with ADHD characterized by prominent hyperactivity-impulsivity, the latent emotion dysregulation variable was related to an increased clustering and local efficiency of the right insula. Thus, in the presence of hyperactivity-impulsivity, clustered network formation of the right insula may underpin emotion dysregulation in young adult ADHD.

## Introduction

Attention-deficit/hyperactivity disorder (ADHD) is a neurodevelopmental disorder characterized by core symptoms of inattention and/or hyperactivity-impulsivity that may persist well into adulthood^1^. Emotion dysregulation, although not a core symptom, is a frequently co-occurring clinical problem^2^. Emotion dysregulation refers to the inability to adequately modulate and control emotions^3^. Its prevalence within the ADHD population changes with age, from 25 to 45% in children to 30–70% in adults, and its co-occurrence is associated with reduced well-being, risky behavior, and social impairments^2^. Especially individuals with ADHD and impulsivity-hyperactivity symptoms often suffer from emotion dysregulation^4^. However, the neural roots of ADHD associated emotion dysregulation remain unclear.

In emotion dysregulation, a distinction between explicit and implicit emotion regulation has been made. Explicit emotion regulation requires conscious effort and is commonly achieved by applying cognitive control and reappraisal strategies. While cognitive control and reappraisal are particularly associated with activity in structures of the ventral attention and frontoparietal network^5^, it is precisely these structures in which deviant—often reduced—activity is frequently found in childhood and adult ADHD studies^1^. Implicit emotion regulation, on the other hand, is an unconscious stimulus-driven process based on experience-based reward estimations. The ventromedial prefrontal cortex and the anterior cingulate cortex have been linked to implicit emotion regulation^5^. With regard to ADHD, it appears that not only structures related to explicit emotion regulation, i.e. structures for cognitive control and reappraisal, are affected, but also those associated with implicit emotion regulation and rather fundamental emotion reactivity processes^6^. Functional connectivity studies consistently showed deviations in structures of the limbic system including the orbitofrontal, ventromedial prefrontal and anterior cingulate cortex in patients with ADHD^7–13^. Also, task-based fMRI studies using emotion perception and processing tasks in individuals with ADHD found evidence for functional abnormalities in the amygdala and insula, possibly indicating increased bottom-up emotional reactivity^14,15^. Task-based fMRI studies related to implicit emotion regulation, i.e., using fear extinction via habituation or emotional Stroop paradigms, which controlled for differences in cognitive control, found ADHD-specific differences in the ventral anterior cingulate and ventromedial prefrontal cortex^16–18^. Given the heterogeneity of ADHD, existing neuroimaging studies, and postulated neurocognitive models, e.g., the dual-pathway model^19^, one might expect ADHD-associated emotion dysregulation to be similarly complex, with both explicit and implicit regulatory processes accounting for it.

While several studies, using task-based as well as resting state fMRI, reported brain activity deviations in structures commonly associated with emotion processing and emotion regulation, few have attempted to directly correlate corresponding activation patterns with emotion dysregulation and none has investigated changes specific to ADHS presentations. Two childhood ADHD studies used seed-based connectivity approaches focusing on the amygdala. They reported associations between high emotion dysregulation scores and reduced negative connectivity with the insula and frontoparietal structures as well as increased positive connectivity with the anterior cingulate cortex^20,21^. Connectivity changes beyond the amygdala have, however, not been investigated, and it remains uncertain whether the reported associations are indeed ADHD- or possibly even ADHD presentation-specific.

To investigate the relationship between emotional dysregulation and functional brain network organization, we used graph theory to analyze fMRI resting-state data from healthy individuals and individuals with ADHD with and without hyperactivity-impulsivity symptoms. We captured the centrality of each brain network node and analyzed whether nodes were highly integrated and connected, either locally towards their direct neighboring nodes or globally with the entire network. Graph theory-based methods have previously been used in ADHD research^10,11,13^ to describe changes in network topology of functional connectivity. These studies show increased local connectivity and efficiency and decreased global integration. It is however unclear how such changes in information processing properties of brain networks relate to emotional deficits. We gauged emotional dysregulation through structural equation modelling (SEM), using a combination of several self and informant scales assessing emotional problems, emotional lability, and conduct problems as well as one experimental task of emotion recognition. SEM is particularly well suited for testing the significance of certain assumed (group-specific) relations while simultaneously estimating latent variables embedded within the relational model. We focused our analysis on nodal topology measures and aimed to identify those brain regions whose local, functional brain network integration specifically contributes to emotion dysregulation in predominately inattentive ADHD (ADHD-I) and ADHD with symptoms of hyperactivity-impulsivity (ADHD-C/H).

We hypothesized that an ADHD-specific association exists between the functional brain network measures for local network integration and emotion regulation. Thereby, we focused on measures of network topology within frontoparietal and limbic brain regions that have previously been associated with emotion regulation and processing. We assumed that the strongest relation would be found in individuals with predominately hyperactive-impulsive ADHD.

## Results

### Sample characteristics

Age and sex did not significantly differ between the ADHD and control group. Participants with ADHD showed higher scores on all four measures of emotional problems and dysregulation. Further, compared to controls, participants with ADHD had a significantly lower IQ, partially showed oppositional defiant disorder (ODD) or conduct disorder (CD) comorbid diagnoses and, in general, more often used stimulant medication. ADHD presentations groups did not significantly differ with regard to age, sex, IQ, the four measures of emotion dysregulation, or stimulant use. The demographic details of the sample are given in Table 1.

**Table 1:** Sample characteristics of the control and ADHD groups as well as of presentation-specific ADHD subgroups.

Group	Controls	ADHD			Controls versus ADHD group comparisons		
		Total	ADHD-I	ADHD-C/H			
	N = 91	N = 56	N = 31	N = 25			
	M ± SD	M ± SD	M ± SD	M ± SD	Test statistic	*ρ*-value	Effect-size
Age (years)	20.2 ± 3.5	19.6 ± 3.5	19.5 ± 3.8	19.7 ± 3.2	T = 1.004	0.317	d = 0.170
IQ (WISC/WAIS)	111.4 ± 13.2	95.9 ± 18.0	98.0 ± 17.7	93.2 ± 18.4	U = 3846	< 0.001	d = 0.501
ADHD, inattention symptoms^a^	0.5 ± 1.1	7.2 ± 1.4	7.2 ± 1.3	7.1 ± 1.6	U = 25.5	< 0.001	d = 0.990
ADHD, hyperactive-impulsive symptoms^a^	0.5 ± 0.9	5.0 ± 2.4	3.4 ± 1.9	7.0 ± 0.96	U = 291.5	< 0.001	d = 0.890
K-10 psychological distress	12.9 ± 3.6	19.7 ± 5.7	19.3 ± 5.8	20.3 ± 5.6	U = 795	< 0.001	d = 0.688
CPRS-R:L emotional lability	44.2 ± 3.3	50.9 ± 9.4	49.5 ± 8.4	52.6 ± 10.5	U = 1412	< 0.001	d = 0.446
SDQ emotional symptoms	1.7 ± 1.6	2.9 ± 2.5	3 ± 2.6	2.8 ± 2.3	U = 1897	0.008	d = 0.255
SDQ conduct symptoms	1.1 ± 1.1	2.1 ± 1.6	2.0 ± 1.6	2.2 ± 1.6	U = 1598	< 0.001	d = 0.373
	n	n	n	N			
Sex (male)	48 (53%)	36 (64%)	20 (65%)	16 (64%)	χ^2^ = 1.443	0.230	φ_c_ = 0.010
Stimulant user (yes)	3 (3%)	30 (54%)	22 (65%)	10 (40%)	χ^2^ = 47.484	< 0.001	φ_c_ = 0.323
DSM-IV ODD (K-SADS)	0 (0%)	13 (23%)	4 (13%)	9 (36%)	χ^2^ = 20.384	< 0.001	φ_c_ = 0.139
DSM-IV CD (K-SADS)	0 (0%)	3 (5%)	1 (3%)	2 (8%)	χ^2^ = 2.658	0.103	φ_c_ = 0.018

Means between groups were compared with independent sample t-tests or Mann–Whitney-U-tests. Frequency distributions were compared with Pearson’s Chi-square (χ^2^)-test. For the CPRS-R:L t-scores are presented, while for the SDQ and K-10 the questionnaire scores are given. ADHD = Attention Deficit/Hyperactivity Disorder; ADHD-C/H: attention-deficit/hyperactivity disorder, combined or predominantly impulsive/hyperactive attention-deficit/hyperactivity disorder; ADHD-I: predominately inattentive attention-deficit/hyperactivity disorder; CD = Conduct Disorder; CPRS-R:L = Conners’ parent rating scale, revised, long version; DSM-IV = Diagnostic and Statistical Manual of Mental Disorders, 4th Edition; HC = Healthy Controls; IQ = Intelligence Quotient; K-SADS = Kiddie Schedule for Affective Disorders and Schizophrenia; K-10 = Kessler Psychological distress scale, NESDA version; N = number of participants; n = number of participants within subgroups; ODD = Oppositional Defiant Disorder; SD = Standard Deviation; SDQ = Strength and Difficulties Questionnaire; WAIS: Wechsler Adult Intelligence Scale; WISC: Wechsler Intelligence Scale for Children.

^a^Combined symptom counts were derived from DSM-IV subscales of K-SADS and the Conners’ rating scales.

### Structural equation models

To identify brain regions in which emotion dysregulation is specifically linked with functional brain network topology as a function of ADHD presentation we combined graph analysis with SEM.

#### Variable selection

In a first step, several tasks, questionnaires, or questionnaire subscales that capture different aspects of emotion dysregulation were examined to identify variables for estimating a latent emotion dysregulation variable. To construct the latent emotion dysregulation variable, we used multi-group confirmatory factor analysis. Significant loadings for the constructed latent variable were found for the emotional distress scores of K-10 (z = 6.008, *p* < 0.001, β = 0.909), SDQ’s emotional symptoms subscale (z = 4.745, *p* < 0.001, β = 0.603), SDQ’s conduct symptoms subscale (z = 3.734, *p* < 0.001, β = 0.546), and CPRS-R:L’s emotional lability subscale (z = 3.044, *p* = 0.002, β = 0.428). These variables were considered for the subsequent SEM, while MINDS-GERT for emotion recognition (z = 0.019, *p* = 0.983, β = 0.002) and ICU for callousness-unemotional traits (z = 1.953, *p* = 0.051, β = 0.163) were discarded. Compared to healthy controls (${\text{s}}_{{{\text{HC}}}}^{2}$ = 0.300) and participants with ADHD-C/H (${\text{s}}_{{{\text{ADHD - C}}/{\text{H}}}}^{2}$ = 0.340), latent variable variance of participants with ADHD-I (standardized variance, ${\text{s}}_{{\text{ADHD - I}}}^{2}$ = 1) was greatest.

#### Three-group structural equation model

**Figure 1:**
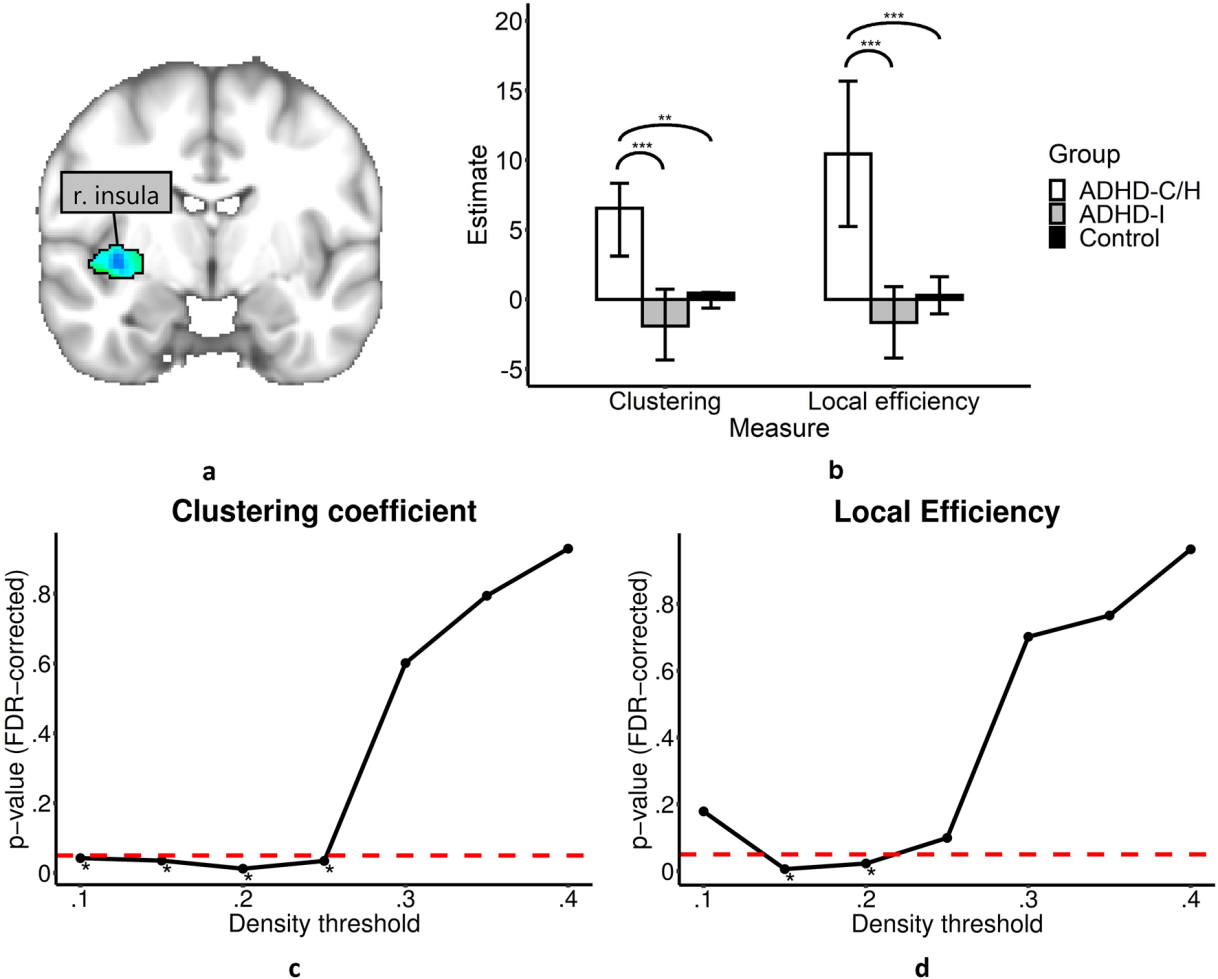
Brain nodes whose nodal network properties are associated with emotional dysregulation in young adult participants with ADHD. Results are shown for the latent variable in relation to local efficiency and clustering measures of the right insula. (**a**) The right insula showed a significant association with emotional dysregulation. Here the underlying parcel is shown. (**b**) Group-specific parameter estimates for the relation between the emotional latent variable and the topology measures (density-integrated) significantly differed between the participants with ADHD-I and ADHD-C/H as well as between participants with ADHD-C/H and controls. 95% confidence interval are displayed. Asterisks give significant group-specific differences (as calculated with χ^2^ difference tests) of the corresponding post-hoc two-group SEM (***p < 0.001, **p < 0.01, *p < 0.05, n.s. *p* > 0.05; ADHD-C/H: attention-deficit/hyperactivity disorder, combined or predominantly impulsive/hyperactive attention-deficit/hyperactivity disorder; ADHD-I: predominately inattentive attention-deficit/hyperactivity disorder). (**c**, **d**) Group-specific differences in the relation between the latent variable and topology measures exist across graphs with different density thresholds (below 0.25). *p*-values of the χ^2^ difference tests are shown. Asterisks signify significance after FDR-correction. The dashed red line represents *p* = 0.05. Figure (**a**) was created using FSLeyes (version 0.22.6, https://fsl.fmrib.ox.ac.uk/fsl/fslwiki/FSLeyes). Figures (**b**, **c**, and **d**) were created with R software (version 3.6.0, https://cran.r-project.org/).

Second, to identify group dependent differences between network topology and the latent variable gauging emotion dysregulation, we used three-group SEM and estimated regression parameters for the group-specific relationship between the latent emotion dysregulation variable and measures of nodal network topology. Pooling over different density levels of connectivity, the SEM revealed group differences in the relation between clustering coefficient as well as local efficiency measures, respectively, and the latent variable for emotion dysregulation in the right insula (see Fig. 1a). For participants with ADHD-C/H the regression parameter estimates of the SEM were greatest, while they were smallest for participants with ADHD-I (see Fig. 1b). Table 2 displays the group-specific z-values for the relation between the latent variable and the right insula’s clustering and local efficiency measures. It also shows *p*-values (uncorrected), and parameter estimates of the completely standardized solution. Further, Table 2 gives model-specific χ^2^, degrees of freedom, *p*-values of the models, goodness-of-fit measures (CFI, SRMR, and RMSEA), *p*-values that are the result of the χ^2^ difference tests between the main models and the corresponding models with fixed regression parameters, and significant pairs of the post-hoc two-group SEM.

**Table 2:** Results of multi-group structural equation model analysis.

Measure	Density	HC			ADHD-I			ADHD-C/H			χ^2^	df	*p*-value	Fit-measures CFI|SRMR|RMSEA	χ^2^ difference test *p*-value (FDR-corr.)	Sig. pairs in Post-hoc χ^2^ difference tests (Bonferroni-corr.)
		z	*p*-value	β	z	*p*-value	β	z	*p*-value	β						
**Right insula**																
Clustering	0.10	− 0.609	0.543	− 0.060	− 0.167	0.868	0.543	3.512	< 0.001	0.754	25.652	21	0.220	0.944|0.880|0.067	0.042	HC vs. ADHD-C/H ADHD-I vs. ADHD-C/H
	0.15	− 0.483	0.629	− 0.246	− 1.453	0.146	− 0.246	3.308	0.001	0.735	26.140	21	0.201	0.939|0.087|0.071	0.035	HC vs. ADHD-C/H ADHD-I vs. ADHD-C/H
	0.20	1.539	0.124	0.172	− 2.110	0.035	− 0.366	3.293	0.001	0.734	23.578	21	0.314	0.970|0.075|0.050	0.012	HC vs. ADHD-I HC vs. ADHD-C/H ADHD-I vs. ADHD-C/H
	0.25	1.256	0.209	0.140	− 2.206	0.027	− 0.392	3.339	0.001	0.733	21.857	21	0.408	0.990|0.063|0.029	0.034	HC vs. ADHD-I HC vs. ADHD-C/H ADHD-I vs. ADHD-C/H
	Integrated	0.820	0.412	0.091	− 1.559	0.119	− 0.279	3.746	< 0.001	0.844	23.472	21	0.319	0.971|0.075|0.049	0.008	HC vs. ADHD-C/H ADHD-I vs. ADHD-C/H
Local efficiency	0.15	− 1.086	0.277	− 0.108	− 1.322	0.186	− 0.215	3.730	< 0.001	0.830	25.535	21	0.225	0.948|0.084|0.066	0.005	HC vs. ADHD-C/H ADHD-I vs. ADHD-C/H
	0.20	1.435	0.151	0.158	− 1.761	0.078	− 0.301	3.297	0.001	0.740	22.604	21	0.365	0.980|0.073|0.039	0.023	HC vs. ADHD-C/H ADHD-I vs. ADHD-C/H
	Integrated	0.424	0.672	0.046	− 1.259	0.208	− 0.222	3.919	< 0.001	0.882	23.693	21	0.308	0.969|0.078|0.051	0.005	HC vs. ADHD-C/H ADHD-I vs. ADHD-C/H

Significant results after FDR-correction are given for the SEM with local efficiency, clustering coefficient of the right insula. All but the last column refer to the three-group SEM with group-specific regression parameter estimates. Group-specific z-statistics for the relation between the latent emotion dysregulation variable and the topology measures, parameter-specific *p*-values (uncorrected), which were obtained by using the quotient of the estimates and their standard error as a test statistic, and β estimates of the completely standardized solution are displayed for significant density-integrated models and the corresponding density-specific models (as calculated with χ^2^-difference tests). The table also displays model-specific χ^2^, degrees of freedom, *p*-values of the models, goodness-of-fit measures (CFI, SRMR, and RMSEA), *p*-values that are the result of χ^2^ difference tests between the main models and the corresponding models with fixed regression parameters, and significant pairs of the post-hoc two-group SEM. Significant two-group differences mainly exist between the ADHD-C/H group and the other two groups.

ADHD-C/H: combined or predominantly impulsive/hyperactive attention-deficit/hyperactivity disorder; ADHD-I: predominately inattentive attention-deficit/hyperactivity disorder; HC: healthy controls.

Only in the ADHD-C/H group were the topology measures of the insula significantly related to emotion dysregulation. The respective models showed satisfactory goodness-of-fit measures. Results were highly significant for the density-integrated models and significant group-specific differences were found across multiple density thresholds below 25% connectivity (see Fig. 1c, d). Results for SEM with clustering coefficients and local efficiency were very similar, as the measures themselves are very similar. Correspondingly, both measures, at the integrated level, showed a correlation of 0.913 for the right insula. Post-hoc two-group comparisons revealed significant group differences between the participants with ADHD-I and ADHD-C/H as well as between participants with ADHD-C/H and controls. For the other nodal topology measures no significant results were found in the right insula.

While not surviving FDR-controlling procedures on the integrated level, SEM with density thresholds between 35 and 40% connectivity revealed group differences in the relationship between eigenvector centrality and the latent variable for emotion dysregulation in the right DLPFC. For participants with ADHD-C/H the regression parameter estimates of the SEM were greatest, while they were smallest for healthy controls. Significant group-specific differences in the relation between topology measures and emotion dysregulation on the uncorrected level occurred for measures of nodes in the right ventromedial prefrontal cortex, frontal pole, right insula, right anterior mid-cingulate cortex, and left insula (all uncorrected *p*-values < 0.01). For none of the other regions examined did we find significant group-specific differences in the relationship between the latent emotion dysregulation variable and measures of nodal network topology.

#### Additional analyses

Robustness of the results was controlled by evaluating the group-specific differences in overall connectivity strength of the individual networks. The analysis of overall connectivity did not reveal significant group differences. Finally, the multi-group SEM analysis was repeated using an alternative parcellation scheme to investigate the robustness of the results with respect to the chosen parcellation*.* The repetition of the main analysis with an alternative parcellation scheme revealed a group-specific difference in the relation between the clustering or local efficiency, respectively, and emotion dysregulation in the right insula. Further significant differences were found for the left insula and the right ventromedial prefrontal corex and frontal pole. The significant frontal pole difference also showed for the density-integrated values and after applying FDR-controlling procedures (clustering coefficient: *p* = 0.047, FDR-corrected; see Supplementary Table S1). The significant right insula result, however, was not revealed using threshold-integrated values but only with a density threshold of 25% connectivity and not after FDR-controlling procedures (clustering coefficient: *p* = 0.033, uncorrected; local efficiency: *p* = 0.030, uncorrected).

## Discussion

Emotion dysregulation is a key component of many psychiatric conditions, including young adult ADHD. We identified properties of functional brain network topology related to a latent measure of emotion dysregulation by using a combination of graph theoretical methods with structural equation modelling. Our results provide evidence that only in individuals with ADHD-C/H emotion dysregulation is accompanied by increases in local efficiency and clustering of the right insula.

Local efficiency and clustering measure how interconnected a node's neighbors are to each other. Increases imply stronger functional connections between structures directly connected to the respective brain region. Here the insula was identified as showing increased connections to its nearest neighbors contributing to emotion dysregulation in ADHD-C/H. The insula integrates interoceptive states with emotional information via connections to other limbic regions such as the amygdala^22^. It plays an essential role in implicit emotion regulation via reciprocal connections with the ventromedial prefrontal cortex and other regions particularly associated with experience-based reward estimations^23^. Throughout brain maturation, functional network formation is characterized by specific patterns of integrative and segregating processes^24^. These processes are also reflected in measures such as the clustering coefficient and local efficiency. Local efficiency in particular has previously been used as a measure of local brain segregation and it was suggested that increased local efficiency goes along with a loss of global brain network integration. For example, it was shown that higher local efficiency is associated with lower global efficiency and reduced performance during cognitive tasks^25^. Prior research consistently provides evidence of delays in the neural maturation of individuals with ADHD^7,26,27^. Increases in the insula’s clustering and local efficiency, i.e. functional connectivity increases between neighboring nodes, may indeed, originate from reduced or delayed network-forming processes. This may negatively affect the efficiency with which the insula performs its integrative tasks and helps to appropriately evaluate emotional stimuli or to facilitate emotion regulation. Our results may reflect ADHD presentation-specific deficiencies in functional network forming processes. At the behavioral level, this could lead to inappropriate behavior and some of the commonly observed emotional problems in ADHD.

The positive association between the functional connectivity of structures directly connected to the insula, i.e. local efficiency and clustering, and emotional functioning in ADHD-C/H was neither found in healthy controls nor participants with ADHD-I. Our results are consistent with expectations, given that this subgroup was shown to be most severely affected by co-occurring emotion dysregulation^4^. Indeed, in the comparison of the different ADHD presentations, the combined type had shown local functional hyperconnectivity in regions associated with emotion processing and implicit emotion regulation, namely the vmPFC^28^. Previously, it was observed that deviations of functional connectivity in ADHD-I occur most clearly in areas of the frontoparietal network, especially the dorsolateral prefrontal cortex, whereas for ADHD-C deviations are most pronounced in areas of the default mode network, i.e. in areas associated with motivation and emotion processing. Since ADHD-I is predominantly characterized by inattention symptoms, it has been suggested that problems in top-down control systems are more likely to underlie this presentation, whereas predominately combined ADHD appears to be more clearly associated with motivation and emotion processing networks^29^. This could not be confirmed with the analysis carried out here. We found no evidence that nodal topology in top-down control regions would be associated with emotion dysregulation in ADHD-I. On the contrary, the right dorsolateral prefrontal cortex showed an ADHD-C/H dependent association between eigenvector centrality and emotion dysregulation. However, these results should be regarded with caution, as they were only seen at two density thresholds. Taken together, the clinical distinction between ADHD presentations with and without hyperactivity-impulsivity symptoms and the associated prevalence differences commonly observed in co-occurring problems such as emotion dysregulation may be reflected in differential deviations of the underlying functional organization of the brain.

Significant results were found for the *right* insula only. While ADHD is not understood as a generally right-lateralized disorder, some functions are more strongly associated with right hemisphere processing^30^. Indeed, the right hemisphere is assumed to take a dominant role in emotion processing. Regarding the regulation of emotions, findings are not as conclusive. However, evidence concerning the insula suggests that processes for integrating interoceptive and emotional/motivational information are rather right lateralized^31^. Note that the left insula yielded—like the dorsolateral prefrontal cortex, right ventromedial prefrontal cortex, frontal pole and cingulate cortex—similar but weaker results, which did, however, not survive correction for multiple comparisons on the integrated level.

The latent variable for emotion dysregulation encompassed self-reported emotional distress, emotional and conduct symptoms and informant-reported emotional lability. Callous-unemotional traits and emotion recognition did not contribute significantly. Their relation to the other emotional measures (i.e., emotional distress and lability) appeared to be rather negligible. This is not surprising as callous-unemotional traits are characterized by deficient affect and lack of empathy, which is a conceptually different aspect of emotion^32^. While emotion recognition is an important prerequisite for the regulation of emotions, adolescents with and without emotion dysregulation have shown similar abilities in recognizing emotions, suggesting again a conceptual difference^33^. Nevertheless, deficits in both are frequent in ADHD, a common occurrence is, however, not imperative^34^.

The novel combination of SEM and graph theory presented here enabled us to reliably assess the functional network topology underlying emotion dysregulation. By using a SEM approach, it was possible to simultaneously determine emotional dysregulation as a latent variable of several emotion questionnaires and assess the group-specific relationships of emotion dysregulation with different measures of nodal brain topology. Previous approaches were limited in focusing on seed based functional connectivity of the amygdala only^20,21^ and using only unidimensional emotional measures. Compared to prior neuroimaging studies, we used a relatively large and well characterized sample of young adult ADHD participants and healthy controls. Also, cases with missing values were excluded from the data set before performing the analyses. Nevertheless, even larger sample sizes may be required in future studies to obtain appropriate power and avoid parameter biases in SEM (see Supplementary Fig. S1). Note however that simple models, as used here, require smaller samples (e.g., about 30 cases for a simple CFA with four indicator variables) and larger samples are often only necessary if missing values exist^35^. The robustness of the findings was further tested by conducting an additional analysis with an alternative parcellation scheme. Even though we were able to replicate the increased local efficiency and clustering in core regions of the implicit emotion regulation network, effects in the insula were weaker and those in ventromedial prefrontal cortex and frontal pole more pronounced. The present study focused on static functional connectivity, not considering the dynamic changes that functional connectivity may show across time, e.g., across the duration of MRI data acquisition. Increasingly, it is suggested that dynamics of functional connectivity are related to behavior and psychopathology^36^, making it a worthwhile target for potential future studies investigating emotion dysregulation in ADHD.

In conclusion, the present study shows a positive relation of the right insula’s clustering and local efficiency with emotion dysregulation in young adult individuals with ADHD-C/H. A similarly strong connection was not found for individuals with ADHD-I or healthy controls. The results suggest ADHD-type specific deficiencies in network forming processes that are associated with emotion processing and its implicit regulation. The commonly observed emotional problems in ADHD may partially be linked to the present findings. Given that emotion dysregulation is present in many other psychiatric disorders it is interesting to note that several of those, i.e. major depressive disorder, bipolar disorder, anxiety disorders and schizophrenia are also associated with changes in the structural and functional connectivity of the insula ^37^, which may represent a worthwhile target area for future treatment efforts.

## Methods and materials

### Participants and procedures

Data were taken from NeuroIMAGE II, the third wave of an integrated genetics-cognition-MRI-phenotype project on ADHD^38^. It includes resting-state fMRI data of 249 individuals with ADHD as well as age- and sex-matched healthy controls. Initial recruitment criteria for ADHD participants were an ADHD combined type diagnosis, availability of one or more siblings, age between 6 and 18 years and availability of subject, sibling, and at least one biological parent for DNA collection. Exclusion criteria (for all participants) were IQ (as measured by Wechsler Intelligence Scale for Children/Wechsler Adult Intelligence Scale) < 70, diagnoses of autism or schizophrenia, and neurological disorders. For controls, it was required that neither they nor any of their first-degree relatives had a prior ADHD diagnosis.

Reassessment of the ADHD diagnosis was established by combining information from the Kiddie Schedule for Affective Disorders (K-SADS)^39^ and parent, teacher, and self-report versions of the Conners’ rating scale (CPRS-R:L, CTRS-R:L and CAARS-R:L)^40–42^. Both the K-SADS and the Conners’ rating scales provide operational definitions of the 18 behavioral ADHD symptoms defined in the DSM-IV. In both, symptoms are subdivided by symptom type, i.e., inattentive symptoms and hyperactive-impulsive symptoms. All diagnostic and phenotypic data was acquired on the same day as the fMRI data. A detailed description of the diagnostic procedures is given by von Rhein et al.^38^.

Participants with a subthreshold ADHD diagnosis (2–4 ADHD-specific symptoms), left-handedness, excessive movement during the scanning, or insufficient quality of rs-fMRI or questionnaire data were excluded. 147 participants were used for the final analysis, including 31 participants with ADHD but without hyperactivity-impulsivity symptoms, i.e., predominantly inattentive ADHD (ADHD-I), 25 participants with ADHD and hyperactivity-impulsivity symptoms (ADHD-C/H), i.e., 21 with combined type ADHD and 4 with predominantly hyperactive-impulsive ADHD, and 91 healthy controls. Demographic information are given in Table 1.

Forty-eight hours prior to testing, stimulant medication use was discontinued. Data acquisition took place at the Donders Institute for Cognitive Neuroimaging, Radboud University Nijmegen, Netherlands. Participants (and their parents when < 18 years old) gave written informed consent for participation. In accordance with relevant guidelines and regulations, ethical approval was granted by the regional ethics board (Centrale Commissie Mensgebonden Onderzoek: CMO Regio Arnhem Nijmegen, ABR: NL41950.091.12). Data analysis was pre-registered using the open science framework (osf.io/rdyp6) ^43^.

### Resting-state fMRI data acquisition and preprocessing

Whole-brain imaging was performed on a 1.5 T Magnetom Avanto (Siemens AG, Erlangen, Germany). BOLD-sensitive resting-state functional volumes were acquired using a T2*-weighted EPI sequence (TR = 1960 ms, TE = 40 ms). Each of the 266 volumes consisted of 37 axial slices of size 64 × 64 (flip angle = 80°, FoV = 224 × 224 mm^2^, voxel-size = 3.5 × 3.5 × 3.0 mm^3^, inter-slice gap = 0.5 mm). T1-weighted high-resolution structural volumes were acquired with an MPRAGE sequence (TR = 2730 ms, TE = 2.95 ms, TI = 900 ms, flip angle = 9°, FoV = 256 × 256 mm^2^, voxel-size = 1.0 × 1.0 × 1.0 mm^3^, GRAPPA 2).

Preprocessing mostly relied on FMRIB algorithms (FSL 5.0.11, https://fsl.fmrib.ox.ac.uk/fsl/) ^44^. The resting-state time series data were skull stripped, realigned to the middle volume of the series, co-registered to the structural T1, and spatially smoothed using a 6 mm full width at half maximum Gaussian kernel (FWHM). ICA-AROMA^45^ was used to account for secondary movement artefacts. Residual noise was further reduced by nuisance regression including a linear trend and average times series measured within the white matter and cerebrospinal fluid. High-pass filtering was conducted at 0.008 Hz. Prior to network analysis, time series were warped to MNI152 space (Montreal Neurological Institute, Montreal, Canada). Root mean squared framewise displacement was calculated. A threshold of 0.25 was applied to exclude 25 participants with extreme movement from further analysis. Root mean squared framewise displacement did not significantly differ between the groups (healthy controls: 0.087 ± 0.071; ADHD-I: 0.128 ± 0.096; ADHD-C/H: 0.111 ± 0.096).

### Graph analysis

For graph analysis we used Python 3.5 (version 3.5.10, https://www.python.org) with NetworkX (version 2.2, https://networkx.org) ^46^. Parcellation of preprocessed time series data was realized using a hemisphere-specific functional brain template with 268 parcels^47^. It was created using graph-theory based parcellation that ensures functional homogeneity within the parcels of the atlas, even across different individuals. The atlas thus minimizes the likelihood that different functional areas lie within a single parcel^47^. For 221 relevant parcels, covered by the MR measurement, subject-specific average intensity time series were calculated. Correlation matrices were created by computing pairwise Pearson’s correlations between the extracted time series. Matrices were Fisher’s z-transformed and absolute values were taken. Absolute value transformation was performed as preprocessing of the present data did not involve global signal regression and as anti-correlations are thought to be functionally relevant^48^. Due to the naturally low density of negative correlations, we refrained from performing specific analysis for positive and negative correlations. To distinguish differences in network density from those of network topology^49^, matrices were binarized based on seven equally spaced density thresholds with a minimum density of 0.10 and maximum density of 0.40^50^. In this range of low to medium network densities, previous studies found significant associations between network topology and ADHD symptoms^10,11,13^. Thus, seven threshold-specific graphs for each subject were investigated in the following graph analysis. Nodal topology measures were calculated for 70 of the 221 nodes. These 70 nodes were chosen based on their association with parcels overlapping with the orbitofrontal cortex, dorsolateral prefrontal cortex, ventromedial prefrontal cortex, anterior cingulate cortex, posterior parietal cortex, insula, ventral striatum, amygdala, and hippocampus as defined by the Harvard–Oxford Brain Atlas by more than 30%. These brain regions have been previously documented to be involved in emotion processing, its regulation, and brain dysfunctions in ADHD^6^.

Here, we captured the centrality of each node and analyzed whether nodes were highly integrated and connected, either locally towards their direct neighboring nodes or globally with the entire network. Thus, our focus is on six nodal measures, that is betweenness, closeness, eigenvector centrality, clustering coefficient, nodal efficiency, and local efficiency, which were used in previous studies on ADHD and showed ADHD-specific deviations^10,11,13^. Betweenness, closeness, and eigenvector centrality are measures that describe the centrality of a node within a network. While betweenness describes how often a node is part of the shortest path between two other nodes, closeness describes how many of the theoretically possible direct connections to other nodes actually exist. Eigenvector centrality also considers the centrality of the node’s direct neighbors. The clustering coefficient of a node describes how strongly its neighboring nodes are interconnected. Efficiency values indicate how directly nodes can be reached from other nodes of the network. In the case of nodal efficiency, this refers to the shortest connection from a particular node to all other nodes, while in the case of local efficiency it refers to the efficiency amongst the nodes adjacent to a particular node of interest. See Supplementary Fig. S2 for a more detailed description of the topology measures. All measures entered into separate SEM models described in 2.4.

**Figure 2:**
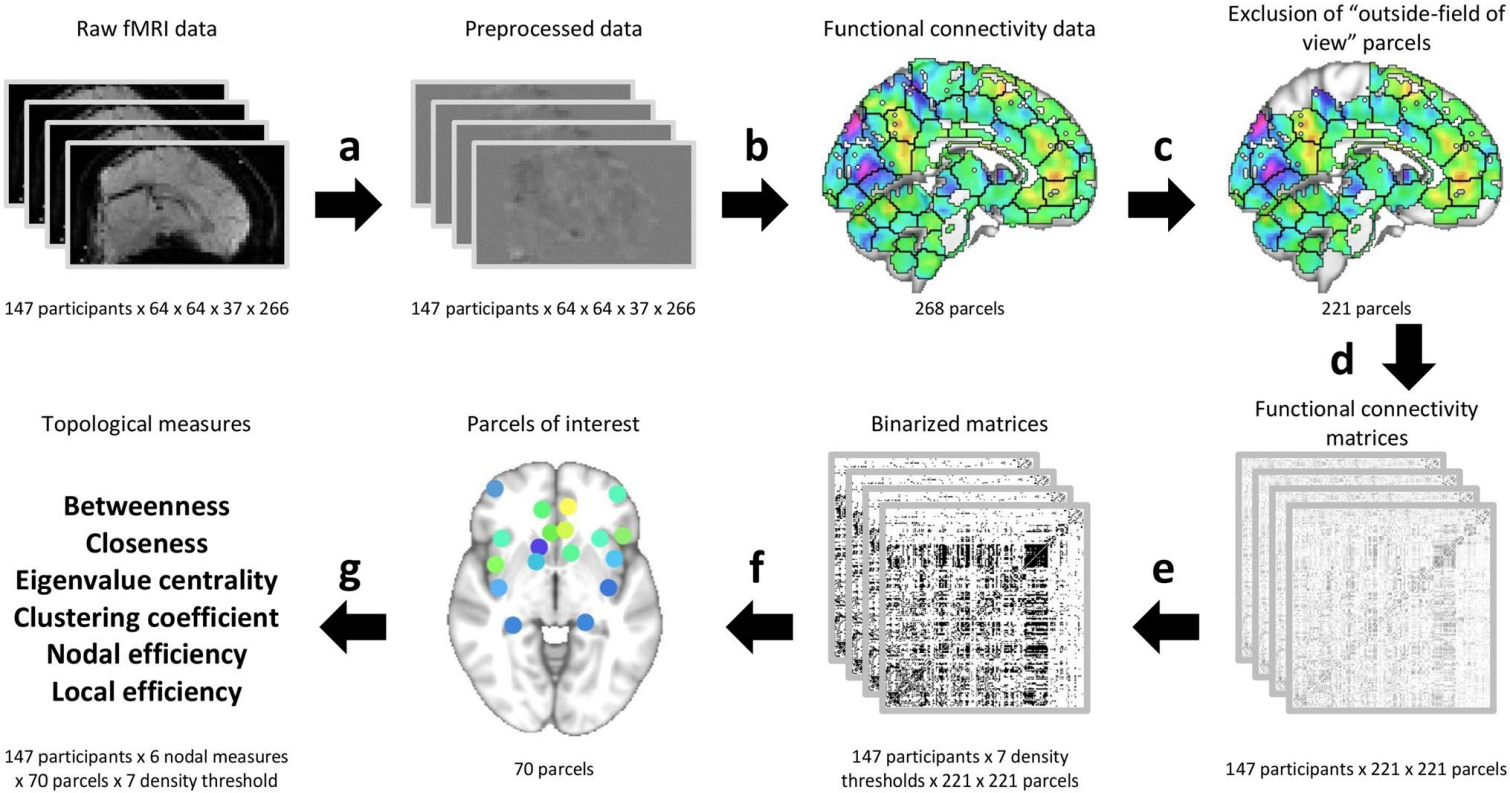
Resting-state functional connectivity analysis pipeline. Preprocessing included skull stripping, co-registration to structural images, realignment to middle volume, spatial smoothing (6 mm FWHM), ICA-AROMA, high-pass filtering (0.008 Hz), nuisance regression, and MNI152-space warping (**a**). Mean activation time series for parcels of functional connectivity template^20,21^ were extracted after exclusion of “outside-field of view” parcels (90% of the time 80% of voxels in a parcel had to have an intensity of > 1000) (**b**, **c**). Individual Fisher’s z-transformed correlation matrices were created (**d**). Each correlation matrix was binarized using 7 different density thresholds (0.10, 0.15, 0.20, 0.25, 0.30, 0.35, 0.40) (**e**). Graphs were created based on binarized matrices and for 70 relevant nodes. Density-integrated as well as threshold-specific topological network measures were calculated (**f**, **g**). Figure was created using FSLeyes (version 0.22.6, https://fsl.fmrib.ox.ac.uk/fsl/fslwiki/FSLeyes) and R software (version 3.6.0, https://cran.r-project.org/).

Density-integrated topology measures were calculated^49^. Differences between populations of weighted networks may be due to the networks’ wiring costs and not the targeted topological features. Density-integration of measures from binarized networks, however, can eliminate cost-related differences and also allow the assessment of topology measures under different density-thresholds. Figure 2 summarizes the functional connectivity and network analysis.

### Statistical analysis and structural equation modeling

#### Variable selection

Statistical analyses were conducted with R software (version 3.6.0, https://cran.r-project.org/) ^51^. Lavaan (version 0.6-8, https://lavaan.ugent.be/) was used for SEM^52^. Prior to SEM, multi-group confirmatory factor analysis was used to identify data suitable for calculation of the latent emotion dysregulation variable. NeuroIMAGE II includes 6 tasks, questionnaires, or questionnaire subscales, respectively, that gauge emotional problems, emotional lability, and associated features: the Kessler Psychological distress scale, NESDA version of K-10^53^ with 10 items for the assessment of emotional problems including anxiety and depression, the strength and difficulties questionnaire subscales (SDQ; five items about anxieties, worries, happiness, and physical symptoms of emotional stress for the emotional symptoms subscale and five items about temper tantrums, compliance, quarrelsomeness, stealing, and lying for the conduct symptoms subscales)^54^, the emotional lability subscale of the Conners’ parent rating scale (CPRS-R:L consisting of three items for unpredictable mood changes, temper tantrums, and tearfulness)^42^, the Inventory of Callous-Unemotional traits (ICU)^55^ (24 items with a callousness and unemotional traits score), and the MINDS Testmanager’s gradual emotion recognition task (GERT)^56^ (accuracy of correct emotion classification).

#### Mediation structural equation model

The latent variable was used to investigate the relationship between functional brain network activity, emotion dysregulation, and ADHD. In the initial, preregistered analysis plan, we intended to use SEM mediation models in which the relationship between topological measures and the emotional latent variable was mediated by ADHD scores (CPRS-R:L) without taking into account the different ADHD presentations. However, those models did not produce good model-fit (as measured by the standard goodness-of-fit measures described below) and had no significant results. One reason may be that the ADHD scores were derived from a parents’ questionnaire for children’s ADHD symptoms (CPRS-R:L) which may be less valid than a clinical diagnosis.

#### Three-group structural equation model

**Figure 3:**
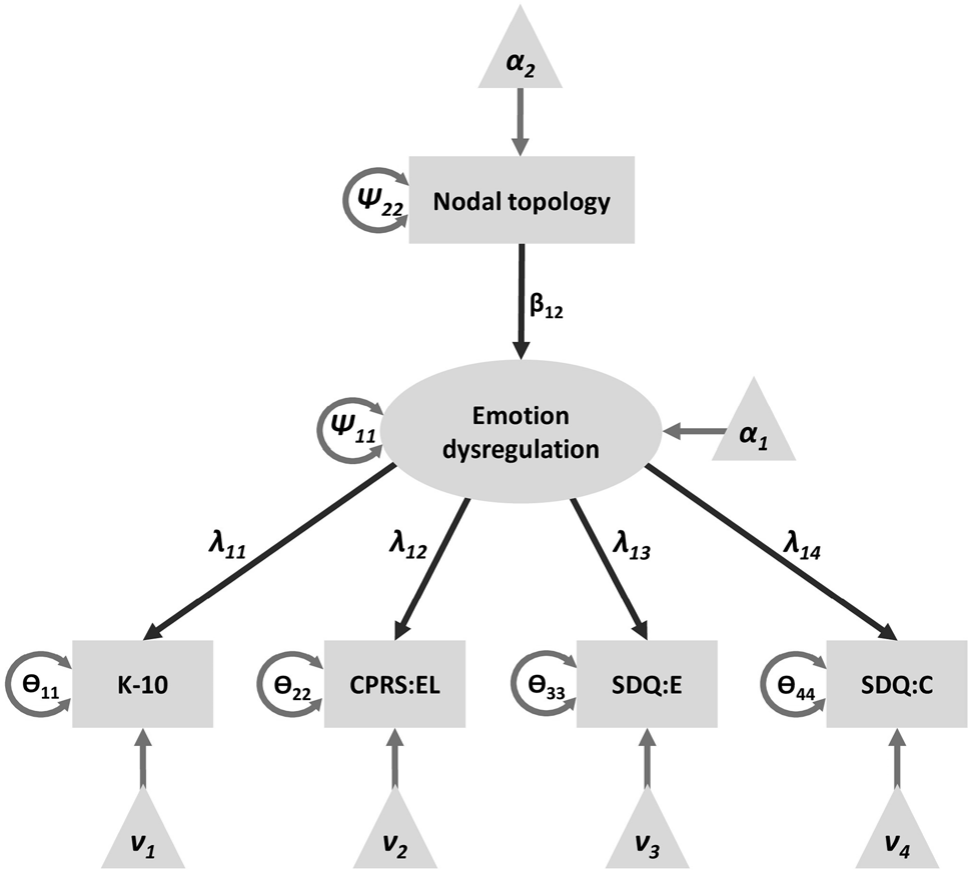
Multi-group structural equation model. Variances (V_1_–V_6_) and intercepts (I_1_–I_6_) are group specific. Loading parameters (p_1_–p_4_) are fixed across groups while the regression parameter (p_5_) is group specific. The model is compared to a model with fixed p_5_; LV: latent variable for emotion dysregulation; K-10: Kessler Psychological distress scale, NESDA version of K-10; CPRS: EL: Conners’ parent rating scale, revised, long version, emotional lability subscale; SDQ: E: Strength and Difficulties Questionnaire, emotional symptoms subscale; SDQ: C: Strength and Difficulties Questionnaire, conduct symptoms subscale. Figure was created using Microsoft PowerPoint 2016 MSO (version 16.0.4266.1001, https://www.microsoft.com/de-de/microsoft-365/powerpoint).

Alternatively, we chose a multi-group SEM approach using the clinical diagnoses. The diagnoses reflect all available diagnostic information and may thus provide more accurate models. We investigated the group dependent differences (i.e., healthy controls, ADHD-I participants, and ADHD-C/H participants) in the association between topology measures and the latent variable gauging emotion dysregulation. Note, that by investigating group-related differences we aimed to identify associations between inter-individual differences in local brain network topology and emotional dysregulation that are specific for individuals with different ADHD presentation. This is different from a mediation model approach (see above) that aims to identify brain regions whose significant correlation with emotional dysregulation only reflects an indirect link via ADHD severity and thus might not directly involve emotion regulation. For each node of interest and each density-specific topology measure, one multi-group model was built. Models consisted of the latent variable, questionnaire scores, associated parameter estimates and variances as well as the nodal topology measure variable with its regression parameter for the latent variable (see Fig. 3). Factor loadings of the latent variable were fixed across groups, latent variable variance was standardized, and to account for between-group mean differences, group-specific intercepts were added. Models were compared with almost identical models, in which however the regression parameters between the network topology variable and the latent emotion dysregulation variable were fixed across groups. Model estimation was conducted using maximum likelihood procedures. To evaluate the significance of the group effects, model-specific χ^2^-values were compared between the two models (χ^2^-difference-test). Following the approach of Ginestet et al.^49^, a model was considered significant if it revealed a significant effect on the density-integrated level (averaged over densities). Subsequently, significance was tested on each density threshold to provide detailed information on whether effects were found with stronger or weaker network connections.

#### Post-hoc analysis, multiple comparison procedures and goodness-of-fit

We used post-hoc two-group SEMs with Bonferroni-corrected *p*-values to investigate pairwise between-group differences. These two-group models resembled the three-group models described above, except that each only considered participants from two of the three groups. Alpha inflation due to multiple comparisons was controlled by the Benjamini–Hochberg false discovery rate (FDR) procedure^57^. The comparative fit index (CFI), standardized root mean square residual (SRMR), and root mean square error of approximation (RMSEA) were calculated to evaluate the models’ goodness-of-fit. Acceptable goodness-of-fit measures should at least be above 0.95 for the CFI, below 0.08 for the SRMR, and below 0.10 for the RMSEA^58^.

#### Additional analyses

To further control the robustness of the results, we evaluated whether group-specific differences in overall connectivity strength of the underlying adjacency matrices exist^59^. Furthermore, an alternative parcellation scheme was used to investigate the robustness of the results with respect to the chosen parcellation approach. For this, we used the AAL atlas^60^, further subdivided with K-mean clustering to adapt the size and number of parcels to that of Finn et al.^47^. Procedures for calculating topology measures and SEM analyses were conducted as described above.

## Supplementary Information

Supplementary Information 1.

## Data Availability

The data are property of the Donders Institute for Cognitive Neuroimaging and are available on request from the corresponding author. The data are not publicly available due to privacy or ethical restrictions. Further information about the NeuroIMAGE Project may be requested via the Project leader Jan K. Buitelaar.
