# Supplementary material for: Functional network topology of the right insula affects emotion dysregulation in hyperactive-impulsive attention-deficit/hyperactivity disorder
Source: Sci Rep. 2021 Jul 22;11:15045. doi: 10.1038/s41598-021-94426-8 (PMC8298497; doi:10.1038/s41598-021-94426-8)
Supplement: Supplementary file 1 — Supplementary Information 1. [file 41598_2021_94426_MOESM1_ESM.pdf]

Supplementary Information

Title:

Functional network topology of the right insula affects emotion dysregulation in hyperactive-impulsive attention-deficit/hyperactivity disorder

Authors:

Tammo Viering, Pieter J. Hoekstra, Alexandra Philipsen, Jilly Naaijen, Andrea Dietrich, Catharina A. Hartman, Jan K. Buitelaar, Andrea Hildebrandt, Carsten Gießing, Christiane M. Thiel

Results of multi-group structural equation model analysis with alternative parcellation scheme

Supplementary table S1: Results of multi-group structural equation model analysis with alternative parcellation scheme. Results are given for the SEM with clustering coefficient measures of the right frontal pole. All but the last column refer to the three-group SEM with group-specific regression parameter estimates. Group-specific z-statistics for the relation between the latent emotion dysregulation variable and the topology measures, parameter-specific p-values (uncorrected), which were obtained by using the quotient of the estimates and their standard error as a test statistic, and β estimates of the completely standardized solution are displayed for significant density-integrated models and the corresponding density-specific models (as calculated with χ^2-difference tests). The group of participants with ADHD-C/H shows the largest β estimates. As compared to the other two groups, all of their estimates indicate significant group-specific relations between the latent variable and the respective topology measure. The table also displays model-specific χ^2, degrees of freedom, p-values of the models, goodness-of-fit measures (CFI, SRMR, & RMSEA), p-values that are the result of χ^2-difference tests between the main models and the corresponding models with fixed regression parameters, and significant pairs of the post-hoc two-group SEM. Significant two-group differences mainly exist between the ADHD-C/H group and the other two groups.

**Measure**	**Density**	**HC**			**ADHD-I**			**ADHD-C/H**			**χ^2**	**df**	**p-value**	**Fit-measures CFI|SRMR|RMSEA**	**χ^2 difference test p-value (FDR-corr.)**	**Sig. pairs in Post-hoc χ^2 difference tests (Bonferroni-corr.)**
		**z**	***p*-value**	**β**	**z**	***p*-value**	**β**	**z**	***p*-value**	**β**						
Frontal pole and ventromedial prefrontal cortex																
Clustering	.15	-1.889	.059	-.217	.304	.761	.057	3.025	.002	.756	38.684	21	.011	.813|.092|.131	.048	- HC vs. ADHD-C/H<br>- ADHD-I vs. ADHD-C/H
	.20	-1.848	.065	-.213	-.037	.971	-.007	3.040	.002	.734	38.995	21	.010	.811|.090|.132	.044	- HC vs. ADHD-C/H<br>- ADHD-I vs. ADHD-C/H
	integrated	-1.863	.062	-.215	.838	.846	-.279	3.029	.002	.748	38.734	21	.011	.812|.091|.131	.047	- HC vs. ADHD-C/H<br>- ADHD-I vs. ADHD-C/H

Notes: **ADHD-C/H**: combined or predominantly impulsive/hyperactive attention-deficit/hyperactivity disorder; **ADHD-I**: predominately inattentive attention-deficit/hyperactivity disorder; **HC**: healthy controls

Results of a priori power analysis for required sample size estimation

Since data collection was already completed when the present analysis was planned, we did not perform any a priori power analysis but used all available data (n=249). We are hesitant to perform a post-hoc power analysis since observed power and p-values are directly related and non-significant p-values naturally correspond to low observed power and vice versa[1]. Reporting power for outcomes already observed is conceptually flawed and analytically misleading[2]. However, we performed an analysis to examine if the existing sample size was close to the sample size estimates of an a priori power analysis for SEM. In SEM, power analyses can be based on χ^2-distributions. If the null hypothesis holds (e.g., model-implied covariance matrix is equal to population covariance matrix), the χ^2-distribution is derived from the degrees of freedom, which in turn can be inferred from the number of manifest variables and free parameters within the model. However, if the alternative hypothesis is true, the distribution additionally depends on a non-centrality parameter. Different non-centrality measures of the effect can be chosen (e.g., RMSEA), all of which depend on the population covariance matrix and the model-implied covariance matrix of the observed variables. One can perform power analyses for SEM with respect to the overall fit of a SEM model or individual parameters. Also, the analyses can be conducted with multi-group SEM[3].

We used the R toolbox semPower to perform a power analysis for SEM[4]. To obtain the population covariance matrix (required for calculating the non-centrality parameter), population values were chosen for all parameters present within the three-group model (medium-sized standardized loadings: .5; group-specific regression parameters: -.5, 0, .5). Note, that we did not estimate these effects from our estimated model since, due to our significant p-values and moderate sample size, our findings might overestimate these effects. Assuming standard values for α and β (α=.05, β=.2) and given a non-centrality parameter of 9.614, a sample size of 37 participants per group was shown to be required in the investigation of the three-group model's regression parameter. This is relatively close to the actual group sizes. However, if we take into account the need for alpha correction due to multiple testing (α=.0007, β=.2), the required sample size changes considerably. Per group, 79 observations are required at a non-centrality parameter of 20.831.

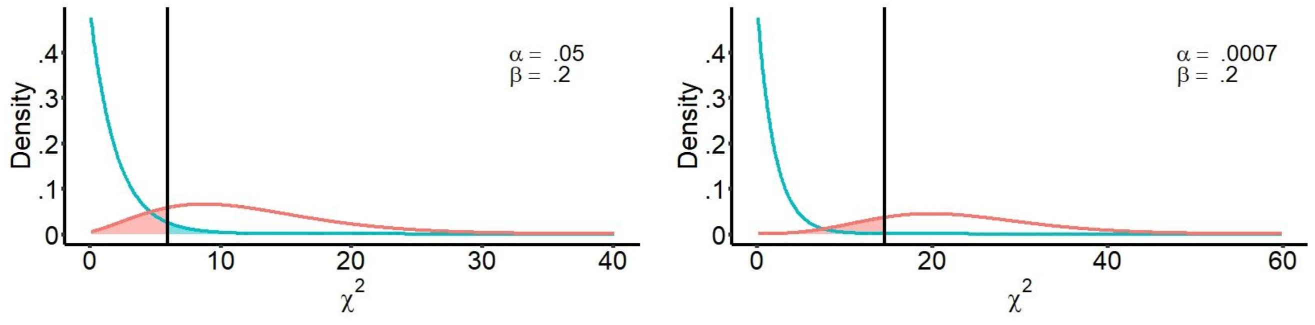

a **b**

Supplementary figure S1: Depiction of central and non-central χ^2-distribution with associated α- and β-areas and the critical χ^2-value. [a] Distributions with required sample sizes of 34 persons per group to obtain α = .05 and β = .2. **[b]** Distributions with required sample sizes of 134 persons per group to obtain α = .0007 and β = .2. Supplementary figures S1a and S1b were created with R software (version 3.6.0, https://cran.r-project.org/).

Description of implemented nodal topology measures with example visualization

Betweenness: The proportion of shortest paths between all pairs of nodes that pass through a given node is described by the centrality measure betweenness. In Supplementary figure S2a, a high betweenness value is given for node 10 with 36% of all paths leading through it ($C_B(10)=0.362$), whereas not a single shortest path leads through node 14 ($C_B(14)=0$). Betweenness centrality of node i in a graph is given as follows [5]:

$$C_B(i) = \frac{1}{(N-1)(N-1)} \sum_{h \neq i \neq j} \frac{\rho_{hj}(i)}{\rho_{hj}},$$

where ρ_{jh} is the total number of shortest paths between node j and h, $\rho_{jh}(i)$ is the number of those paths that go through i and N is the number of nodes in the graph.

Closeness: Closeness is based on the average shortest path length, where the shortest path length is the minimum number of edges that have to be traversed to travel from one node to another. To obtain closeness, the inverse of the average shortest path length is taken, resulting in a high closeness value for nodes with low average shortest path lengths and vice versa. In Supplementary figure S2b, high values can be observed for the most central nodes ten ($C_C(10)=0.556$) and 11 ($C_C(11)=0.613$), while particularly noncentral nodes have very low values. Closeness centrality of node i in a graph is given as follows [5]:

$$C_C(i) = \frac{N-1}{\sum_{j \neq i} l_{ij}},$$

where l_{ij} is the shortest path length between nodes i and j and N is the number of nodes in the graph.

Eigenvector centrality: Node degrees are the basis of eigenvector centrality. The degree of a node describes the number of edges connecting the node to all other nodes of the network. Based on this, the eigenvector centrality considers not only the degree of the node in question but also those of its neighbors. The required information is contained in the graph's adjacency matrix and the eigenvector centrality of node i within a network is given by entry i of the eigenvector belonging to the largest eigenvalue of the adjacency matrix. Unlike betweenness and closeness, eigenvector centrality specifically emphasizes the centrality of the 11 ($C_E(11)=0.357$). node (see Supplementary figure S2c), since not only this node but also its neighboring nodes have high node degrees. Eigenvector centrality of node i in a graph can be derived from the eigenvector equation [5]:

$$C_E(i) = \frac{1}{\lambda_1} \sum_{j=1}^{N} A_{ij} x_j,$$

where A is the adjaceny matrix of the graph, λ_1 is the largest eigenvalue, x is the associated eigenvector and N is the number of nodes in the graph.

Clustering coefficient: The clustering coefficient of a node is the fraction of its neighbors that are also connected to each other. It thus describes how close its neighbors are to being a clique, i.e. a complete graph. For several nodes of Supplementary figure S2d (namely nodes 9, 12, 17), maximum clustering coefficient values are given, indicating that they are located in a highly interconnected cluster. At the same time, however, it is also evident that a high clustering coefficient can arise from a node only having very few connections (node 14). In contrast, the centrally located node 10 has seven connections and correspondingly 28 connections between its neighbors are possible. Only eight of these actually exist and its clustering coefficient accordingly is $Cl(i)=0.285$. The clustering coefficient of node i is defined as[5]:

$Cl(i) = \frac{2t_i}{k_i(k_i-1)}$,

where t_i denotes the number of edges connecting the k_i neighbors of node *i*.

Local efficiency: The local efficiency is another measure based on shortest path lengths. To calculate it, only those nodes are considered that are directly connected with a certain node of interest. For all node pairs within the resulting subgraph, the reciprocal of the shortest path length is calculated. Subsequently, the mean of the reciprocals is taken to determine the local efficiency. The conceptual similarity to the clustering coefficient is also reflected in the results of the example graph. Values that are high in one topology measure are also high in the other and vice versa (see Supplementary figures S2d & S2e). Local efficiency of node *i* is obtained as follows[5]:

$E_{loc}(i) = \frac{1}{k_i(k_i-1)} \sum_{j,h=1}^{k_i} \frac{1}{l_{jh}}$,

where l_{jh} is the shortest path length between two nodes *j* and *h* directly connected to node *i* and k_i is the number of direct neighbors of node *i*.

Nodal efficiency: To calculate the nodal efficiency, the reciprocals of the short path lengths between a target node and all other nodes of the graph are required. To determine the nodal efficiency of the target node, the average of the reciprocals is taken. The nodal efficiency indicates how well a given region is integrated into a network via its shortest paths. This is especially the case for node 10 ($E_{nod}(10)=0.684$), but also node 11 ($E_{nod}(11)=0.675$; see Supplementary figures 2f). Nodal efficiency of node *i* is obtained as follows[5]:

$E_{nod}(i) = \frac{1}{N-1} \sum_{i \neq j} \frac{1}{l_{ij}}$,

where l_{ij} is the shortest path length between nodes *i* and *j* and *N* is the number of nodes in the graph.

To obtain more details about the calculation of graph topology measures, we refer to the fundamental literature by Fornito, Zalezky, and Bullmore[5].

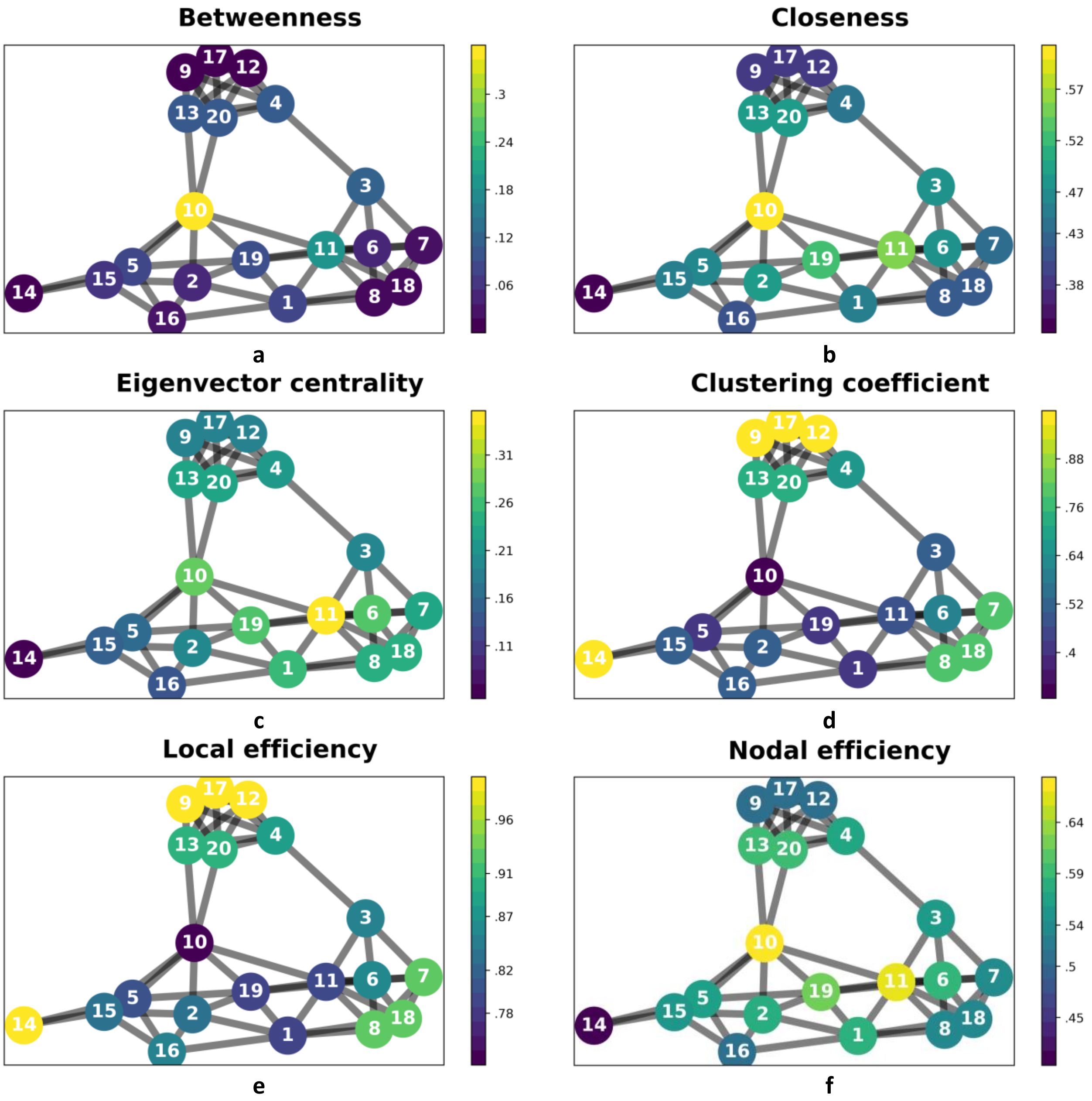

Supplementary figure S2: Example graph with subplots visualizing different nodal topology measures. The example graph was created with the random geometric graph function from NetworkX (version 2.2, https://networkx.org)[6]. The values of the node-specific topology measures are indicated by the nodes' colors. Supplementary figure S2 was created using Python (version 3.5.10, https://www.python.org) and the associated library Matplotlib (version 2.2.3 , https://matplotlib.org/) .

Literature

1. Hoenig, J. M. & Heisey, D. M. The Abuse of Power: The Pervasive Fallacy of PowerCalculations for Data Analysis. *Am. Stat.* **55**, 19–24 (2001).

2. Zhang, Y. *et al.* Post hoc power analysis: is it an informative and meaningful analysis? *Gen. psychiatry* **32**, e100069 (2019).

3. Jak, S., Jorgensen, T. D., Verdam, M. G. E., Oort, F. J. & Elffers, L. Analytical power calculations for structural equation modeling: A tutorial and Shiny app. *Behav. Res. Methods* 1–22 (2020). doi:10.3758/s13428-020-01479-0

4. Moshagen, M. & Erdfelder, E. A New Strategy for Testing Structural Equation Models. *Struct. Equ. Model. A Multidiscip. J.* **23**, 54–60 (2016).

5. Fornito, A., Zalesky, A. & Bullmore, E. T. *Fundamentals of Brain Network Analysis*. *Fundamentals of Brain Network Analysis* (2016). doi:10.1016/C2012-0-06036-X

6. Hagberg, A., Schult, D. A. & Swart, P. J. Exploring Network Structure, Dynamics, and Function using NetworkX. (2008).
